# Identification of the xenograft and its ascendant sphere-forming cell line as belonging to EBV-induced lymphoma, and characterization of the status of sphere-forming cells

**DOI:** 10.1186/s12935-019-0842-x

**Published:** 2019-05-06

**Authors:** Evgeniya V. Dolgova, Daria D. Petrova, Anastasia S. Proskurina, Genrikh S. Ritter, Polina E. Kisaretova, Ekaterina A. Potter, Yaroslav R. Efremov, Sergey I. Bayborodin, Tatiana V. Karamysheva, Margarita V. Romanenko, Sergey V. Netesov, Oleg S. Taranov, Aleksandr A. Ostanin, Elena R. Chernykh, Sergey S. Bogachev

**Affiliations:** 1 Institute of Cytology and Genetics, Siberian Branch of the Russian Academy of Sciences, 10 Lavrentiev Ave., Novosibirsk, 630090 Russia; 2 Novosibirsk State University, Novosibirsk, Russia; 3 State Research Center of Virology and Biotechnology “Vector”, Koltsovo, Novosibirsk, Russia; 4 Research Institute of Fundamental and Clinical Immunology, Novosibirsk, Russia

**Keywords:** B-cell lymphoma, Lymphoblastoid cell line, Mesenchymal stem cells, Clonotypic B cell, TAMRA-labeled DNA probe

## Abstract

**Background:**

We have characterized the human cell line arised from the Epstein–Barr virus (EBV) positive multiple myeloma aspirate subjected to the long-term cultivation. This cell line has acquired the ability to form free-floating spheres and to produce a xenograft upon transplantation into NOD/SCID mice.

**Methods:**

Cells from both in vitro culture and developed xenografts were investigated with a number of analytical approaches, including pathomorphological analysis, FISH analysis, and analysis of the surface antigens and of the VDJ locus rearrangement.

**Results:**

The obtained results, as well as the confirmed presence of EBV, testify that both biological systems are derived from B-cells, which, in turn, is a progeny of the EBV-transformed B-cellular clone that supplanted the primordial multiple myeloma cells. Next we assessed whether cells that (i) were constantly present in vitro in the investigated cell line, (ii) were among the sphere-forming cells, and (iii) were capable of internalizing a fluorescent TAMRA-labeled DNA probe (TAMRA+ cells) belonged to one of the three types of undifferentiated bone marrow cells of a multiple myeloma patient: CD34+ hematopoietic stem cells, CD90+ mesenchymal stem cells, and clonotypic multiple myeloma cell.

**Conclusion:**

TAMRA+ cells were shown to constitute the fourth independent subpopulation of undifferentiated bone marrow cells of the multiple myeloma patient. We have demonstrated the formation of ectopic contacts between TAMRA+ cells and cells of other types in culture, in particular with CD90+ mesenchymal stem cells, followed by the transfer of some TAMRA+ cell material into the contacted cell.

**Electronic supplementary material:**

The online version of this article (10.1186/s12935-019-0842-x) contains supplementary material, which is available to authorized users.

## Background

Multiple myeloma (MM) belongs to incurable lymphomas. This is B cell cancer characterized by uncontrolled proliferation of malignant plasmacytoid B cells, their clonal expansion into the bone marrow space, and synthesis of a monoclonal immunoglobulin, paraprotein, being in excess in the bone marrow, blood plasma, and urine. The typical clinical MM manifestations are anemia, bone pain (associated with lytic bone destruction), renal failure, hypercalcemia, and an increased risk of infectious diseases [1, 2].

Any attempt to transfer MM cells into the culture confronts a principal problem associated with the possible persistence of the Epstein–Barr virus (EBV) in the patient’s organism. This virus is known to infect B cells. Viral infection corrupts the mechanisms regulating the proliferation in these cells, as well as inhibits the apoptosis. All these dysfunctions lead to the switching of B cells to a malignant state. Nevertheless, the infection is never associated with the integration of viral DNA into the host genome. The virus-mediated immortalization is accepted to be associated with the peculiarity of synthetic processes in the latent “0”, “I”, “III” stages of the virus propagation [3].

It is established that, in the absence of immune control (e.g. during in vitro culturing or transplantation into immunodeficient mice), EBV-infected B cells demonstrate the high rate proliferation. It is also known that the proliferation rate of EBV-infected B clone is significantly higher than that of clonotypic MM cell (doubling time 36–48 h vs. 108–144 h), and thus it has selective growth advantages in the absence of T8 EBV-specific cytotoxic lymphocytes. Long-term cultivation of such mixed cultures leads to the displacement of MM cells and the consequent domination of one or more of EBV-positive clones. With further cultivation, the only one dominant clone capable of producing an idiotypic immunoglobulin remains. Such cell lines are known as EBV-positive B lymphoblastoid cell lines (EBV+ LCLs) [4–6]. EBV-mediated in vitro immortalization of B cells provides a model for investigation of EBV-induced lymphomagenesis in human, such as Burkitt lymphoma or some cases (30–40%) of Hodgkin’s lymphomas [3]. Thereby, despite their “artificial” origin, EBV-induced cell lines could be a valuable object for modeling and investigation of the events occurring in vivo.

Aspirate taken from a patient to obtain a MM cell culture is inevitably contaminated with B lymphocytes. If a patient is being infected with EBV, the virus is localized in B lymphocytes. This is the case, when the culture will inevitably be contaminated with infected B cells, which gradually supplant the MM cells during the cultivation process. As a result, the EBV+ LCL will be obtained. Thus, in experimental hematology it is strictly accepted that a cell line obtained from a patient with MM can not be definitively considered a MM cell line [6].

In our study [7], we have characterized the properties of cells derived from a bone marrow aspirate of a MM patient with regard to their capability of forming free-floating spheres. Primary free-floating spheres were shown to contain cells with the characteristic B cell surface marker CD20. The spheres contained 1–3% of cells with inherent capability of internalizing a TAMRA-labeled double-stranded DNA probe [7–14]. These undifferentiated cells, together with a group of floating TAMRA-negative cells, constituted a sphere-organizing center, inducing repeated aggregation of cells into a spherical 3d structure. Spatial interaction of TAMRA-positive cells with other cells of the spheres is proposed to be due to their ability to secrete certain sets of cytokines specific for each types of these cells. The conducted grafting experiments established that these cells have to be organized into spheres for successful grafting. Only these cell structures, but not single cells, provided the xenograft development upon inoculation into NOD/SCID mice [7].

In this study, we pursued two objectives.

The absolute majority of cells were CD20 positive, which is exclusive hallmark of EBV+ LCL. Moreover, during the cultivation process, this cell line has acquired the ability to form distinctive spheres, which is another hallmark of EBV+ LCL. Thus, imprimis, it was necessary to clarify the B-cellular origin of the investigated immortalized cell line. It was also necessary to prove the relation of the developed graft [7] to the cell line used for transplantation.

The second objective was to evaluate a possible relation of TAMRA-positive cells to one of the three types of bone marrow stem cells of a MM patient, namely CD34 hematopoietic progenitors, clonotypic B cells (regardless of their relationship to either MM or EBV-transformed B-cells), and mesenchymal stem cells (MSCs).

## Materials and methods

### Cell line

The cell line analyzed in this study was the same as that previously reported [7].

### Pathomorphology of tissues and organs

Organs fragments were fixed in 4% formaldehyde, dehydrated in graded series of alcohols, cleared in xylol, and embedded in paraffin. Five micrometer paraffin sections were stained with hematoxylin and eosin. An AxioImager ZI microscope (Zeiss) was used for analysis.

### DNA isolation from xenografts and cells in vitro

To obtain a solid xenograft, 10^6^ of cells were engrafted subcutaneously into the scapula area of NOD/SCID mice. The procedures were carried out in the SPF Animal Facility, Institute of Cytology and Genetics SB RAS. All experiments with mice were approved by the Inner-institute Bioethics Committee (Institute of Cytology and Genetics SB RAS) and carried out in accordance with Guide for the Care and Use of Laboratory Animals. A piece of xenograft tissue was dispersed into individual cells using a homogenizer. A cell suspension was washed several times with αMEM medium (Gibco), passed through a 70 μm cell strainer (BD Bioscience). Cells were precipitated by centrifugation at 400*g* for 5 min and resuspended in PBS supplemented with 50 mM EDTA and 0.1% SDS. In the case of cell culture, cells were pelleted by centrifugation, and the same buffer (PBS/50 mM EDTA/0.1% SDS) was added to the cell pellet. Then, in both cases, the resulting lysate was supplemented with 200 μg/mL of proteinase K (Fermentas, Life Sciences) and incubated at 58 °C for 30 min. After proteinase treatment, the extraction with an equal volume of phenol/chloroform was performed; DNA was precipitated, and dissolved in mQ H_2_O. The DNA concentration was measured using a Qubit 2.0 fluorometer (Invitrogen).

### Sequencing of VDJ locus from DNA isolated from the xenograft and initial culture

The DNA isolated from xenograft samples and cells in vitro was amplified in a standard PCR using the following primers [15, 16]:

JH: 5′-ACCTG-AGGAG-ACGGT-GACCA-GGGT-3′
FR1c: 5′-AGGTG-CAGCT-GSWGS-AGTCD-GG-3′
Fr3c: 5′-GACAC-GGCCG-TGTAT-TACTC-3′
FR2b: 5′-GTCCT-GCAGG-CCCCC-GGAAA-AAGTC-TGGAG-TGG-3′

The resulting 500 bp fragment was purified from agarose (DNA cleaning kit, Medigen) and cloned into the pBlueScript plasmid at the *Eco*RV restriction site. The resulting vector was transformed into electrocompetent cells XL Blue MRF’, and the cells were seeded on agar supplemented with X-Gal (20 μg/mL), IPTG (1 mM), and Amp (25 μg/mL). The plasmid DNA was isolated from ~ 30 white colonies, and the insert was sequenced on an ABI3130xl 16-capillary automatic DNA sequencer in accordance to Applied Biosystems protocols at the Interinstitutional DNA Sequencing Center of the Siberian Branch of the Russian Academy of Sciences. Big Dye 3.1 (Applied Biosystems, USA) was used as a dye. Sequences were analyzed using the Vector NTI software.

### Labeling of human Alu repeat DNA and of the 500 bp B clone DNA fragment with TAMRA or Flu

TAMRA labeling of *Alu*-repeat DNA was performed exactly as described in a study by [12]. The same protocol was used for Flu-5′-dUTP labeling.

### Quantitative PCR and calibration curves

DNA molecules were quantified by the real-time PCR using a SYBR Green PCR Master Mix (# 4309155, Applied Biosystems, UK). To generate quantitative PCR (qPCR) calibration curves, JH/FR3c primers (the sequence is specified in paragraph above) as well as specific primers for human *Rplp0* gene locus or for mouse prostaglandin E receptor 2 (*PTGER2*) gene locus were used (sequences are indicated below); varying concentrations of the 500 bp B clone DNA, total human DNA, and total mouse DNA each were added to the reaction. Each concentration was tested in triplicate.

Rplp0 for: 5′-TCATC GTAAG TGCAG GGTGG-3′
Rplp0 rev: 5′-TGCCT GAGCC CGTTT ATCTG-3′
PTGER2 for: 5′-CCTGC TGCTT ATCGT GGCTG-3′
PTGER2 rev: 5′-GCCAG GAGAA TGAGG TGGTC-3′

A linear plot of Ct vs DNA content was constructed using the Microsoft Excel software. Template DNA (usually, 100 ng, if not specified) was added to each qPCR reaction. The number of B clone copies present in the template DNA was quantified using the calibration curve slope; human vs. mouse material in the NOD/SCID mouse xenograft was quantified using the Step One software v2.3. All real-time PCR experiments were performed as triple tests repeated several times (from 3 up to 9 times in different experiments) using a Step One Real-Time PCR System (Applied Biosystems).

### Cell surface phenotype analysis

Cells were immunostained with monoclonal antibodies against different cell surface markers (BD Biosciences, San Jose, CA). Samples were analyzed using a FACSCalibur cytometer and CellQuest software (BD Biosciences).

### Dual analysis of CD90 surface expression and TAMRA-Alu incorporation by cells in vitro

A total of 10^6^ cells were incubated in 200 μL of αMEM with 0.2 μg of TAMRA-labeled *Alu* DNA at room temperature for 1 h. Then, APC-conjugated CD90-specific antibodies (Sony Biotechnology) were added to the cell suspension (1:500). Next, the cell suspension was either spun on glass slides using a cytospin (1000 rpm for 1 min) or analyzed directly in the culture. In the first case, cells were layered with a drop of Antifade DABCO (Sigma-Aldrich) supplemented with 0.5 μg/mL DAPI (Sigma-Aldrich) and covered with a coverslip. The analysis, including video, was performed using a LSM 780 NLO (Zeiss) confocal fluorescence microscope and ZEN software at the Collective Use Center for Microscopy of Biological Objects, the Siberian Branch of the Russian Academy of Sciences.

### FISH

A fluorescently-labeled DNA probe (prepared as described above) was dissolved in 30 μL of hybridization buffer (2× SSC, 50% formaldehyde, 10% dextran sulfate, 1% NP). About 1–1.5 × 10^6^ cells were spun onto glass slides using a cytospin, then fixed in a methanol:glacial acetic acid mixture (3:1), and air dried. Samples were placed into 2% paraformaldehyde for 10 min and then washed twice with PBS. Cells were permeabilized with 0.5% Triton X-100 for 10 min and washed with PBS. Next, samples were treated in series of ethanol baths (70, 80, and 100%) and air-dried. Five microliters of a DNA probe (~ 0.15 μg/mL) were dropped on each glass slide; the latter was covered with coverslips and sealed with rubber cement. Preparations were denatured and then kept in the wet hybridization chamber overnight. Further, the samples were incubated with 1× SSC at 60 °C for 5 min, then with 4× SSC + Np40 at 37 °C for 10 min. Samples were washed with deionized water and treated in series of ethanol baths. Then, samples were dried in the dark at 37 °C, supplied with Antifade DABCO supplemented with 0.5 μg/mL DAPI, and covered with a coverslip. Fluorescence signals were detected on an Axioskop 2 Plus fluorescence microscope (Zeiss) using the ZEN software.

### Characterization of the cell line obtained from the multiple myeloma patient

The analysis to characterize the reported cell culture has been ordered to and completed in the certified laboratory “INVITRO” (LLC “INVITRO” , medical license LO-43-01-002895 from 01.11.2018, https://www.invitro.ru).

### Detection of EBV

Total DNA from the cells of the reported line was isolated. EBV-specific DNA was detected by PCR with specific primers (assay #351URO).

### Detection of the paraprotein in culture medium

Cells were sedimented at 400*g* for 5 min and washed twice with serum-free αMEM medium. Approximately 5 million cells were resuspended in this serum-free medium and further incubated in the CO_2_-incubator for 48 h. After this, cells were sedimented at 12,000*g* for 15 min and supernatant has been collected for the following electrophoretic assay and immunofixation with the set of antisera (to IgG, IgA, IgM, kappa and lambda chains) followed by densitometry and estimation of the M-component content (assay #4051). Total protein content in the supernatant turned out to be too low to be detected by the used approach.

### FISH analysis of the cultured cells genotype

Cells were sedimented at 400*g* for 5 min and washed twice with PBS. Approximately 10 million cells were fixed with methanol:acetic acid (3:1). Cells were FISH assayed for rearrangement in the IGH/14q32 gene locus, deletion/amplification of the CKS1B/1q21 and CDKN2C/1p32 genes loci, and deletion of the DLEU/13q14.2, LAMP/13q34 and TP53/17p13 genes loci (assay #5.121). For each issue, 200 nuclei have been analyzed.

### FICTION

About 5 × 10^5^ cells were spun onto glass slides using a cytospin, air-dried, fixed with acetone for 10 min, and air-dried again. Samples were incubated with APC-conjugated CD90 antibodies (Sony Biotechnology) and fixed by addition of a cold methanol:glacial acetic acid mixture (3:1) for 10 min, followed by treatment with 1% paraformaldehyde for 1 min. Further, the above described FISH protocol was used.

### Transmission electron microscopy analysis

For electron microscopic examination, cells were precipitated by centrifugation (200*g* for 5 min) and the pellet was further fixed with 1% osmic acid solution, dehydrated with a standard technique in increasing concentrations of ethyl alcohol and acetone and embedded in Epon–Araldite mixture. Ultrathin sections were prepared on the Reichert-Jung microtome (Austria), contrasted with uranyl acetate and lead citrate. The sections were examined in the JEM 1400 electron microscope (Jeol Ltd., Tokyo, Japan); images were taken with the Jeol integrated digital camera and the Veleta side output digital camera (Olympus Soft Imaging Solutions GmbH, Munster, Germany). The images were processed and analyzed using the software iTEM (Olympus Soft Imaging Solutions GmbH).

## Results

### Characterization of the investigated cell line as EBV+ LCL

#### Pathomorphological analysis of bone-cartilage tissues from the mouse with subcutaneously grafted cells

**Fig. 1:**
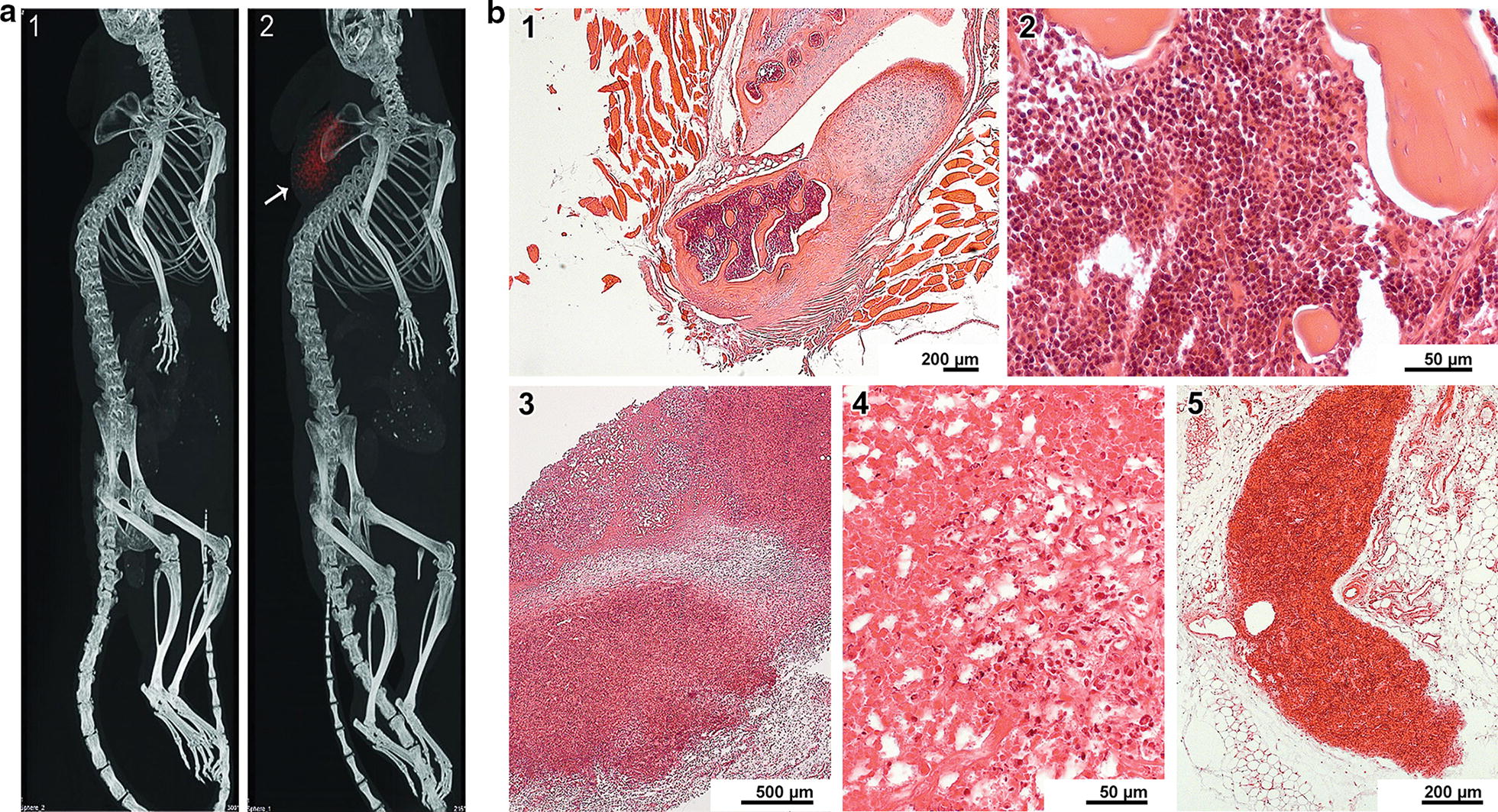
Pathomorphological analysis of MM grafted NOD/SCID mice. **a** Computed tomography images of NOD/SCID mice. 1—Grafting of a suspension of cells that are not involved in spheres. 2—Grafting of cells in the form of sphere aggregates. A subcutaneously grafted tumor (spheres) is indicated by red color and the arrow. **b** Pathomorphological analysis of fragments of animal bones, tissues, and organs. 1—An osteocartilaginous junction surrounded by muscle fibers. No pathological changes. 2—The bone marrow with a normal cell composition. 3—A necrotized tumor conglomerate. 4—Multiple neutrophilic granulocytes, erythrocytes, and a cellular detritus in the tumor necrosis site. 5—A tumor lesion in adipose tissue. Numerous blood vessels with free lumens are seen. No inflammatory reaction

Several studies have demonstrated that intravenous inoculation of human MM clonogenic cells in SCID mice results in the development of the typical CRAB features of MM pathology in the experimental animals [1]. To reveal clinical features of the original disease (MM) in the mouse with an induced xenograft, a pathomorphological analysis of tissues, skeleton, and organ fragments of the animal was performed. The results are shown in Fig. 1.

The most typical pathological manifestation of MM is the complex of CRAB symptoms, and the most significant manifestation is the lysis of bone tissues [1]. Analysis of samples containing bone-cartilaginous lesions (11 in total) revealed tumor tissue in 3 of them. In two cases, large foci of tumor growth were found in the vicinity of muscle, adipose tissue, and blood vessels. In both cases, the process was accompanied by necrosis in tumor nodes (one of the samples demonstrated pronounced inflammation with abundant polymorphic cellular infiltration). At the same time, intact foci of tumor growth without any signs of necrosis and/or inflammation were observed slightly aside, in adipose tissue. In the third sample, very few tumor cells were occasionally scattered in bones, cartilages, muscles, and adipose tissue. There were no signs of their large-scale invasion into bone and cartilaginous tissues. The lack of integration of cancer cells into bone tissue and the apparent absence of the pathological lysis of bone and cartilage structures is not typical of the development and clinical manifestation of MM.

This pathomorphological analysis findings could be due to the grafting procedure was performed subcutaneously. Upon such a grafting procedure, a MM tumor cell is incapable of immediate expansion into the medullary space, and when the cell reaches the bone marrow compartments, it does not have stromal support. For these reasons, MM tumor cells colonize any available anatomical structures, which are not typical of MM, but at the same time, are most favorable for the survival of these cells, instead of the regular bone marrow niches. Another explanation could be that the resulting xenograft is not MM, but another form of B cell lymphoma, such, in particular, as EBV+ LCL.

#### Cell culture contamination with EBV

The “principal and ultimate” criterion of whether the obtained cell line belongs to either MM or EBV+ LCL is the presence or absence of EBV contamination. MM cell lines in vitro are never infected with EBV, while EBV+ LCLs are always EBV-positive [4–6]. The conducted qualitative PCR with specific primers proved the contamination of the investigated cell line with Epstein–Barr virus.

#### Monoclonal immunoglobulin expression in cells of the investigated line

The additional “moderate” criterion of whether the obtained cell line belongs to either MM or EBV+ LCL is the expression of specific monoclonal immunoglobulin, which can be detected in the culture medium. “Moderateness” of this criterion is due to that MM cells do not always (necessarily) produce the paraprotein, while the dominating B clone, which completely substitutes MM cells in the EBV-contaminated culture over the long-term cultivation, acquires the ability to produce a specific monoclonal immunoglobulin [4–6].

To detect whether the obtained cell line produced the paraprotein, cells were cultivated in the complete tissue culture medium until the spheres had been formed. Further, cells were washed out of the serum-containing culture medium, resuspended in ɑMEM and subjected to incubation under regular conditions (37 °C, 5% CO_2_) for additional 48 h. To detect and identify the protein as belonging to a certain class of immunoglobulins, the serological analysis was carried out. The conducted analysis revealed no expression of the class-specific immunoglobulin in the investigated cells.

#### Chromosomal analysis of the reported cell line

EBV-induced B-cellular lymphoblastosis at the initial stages of development displays no chromosomal aberrations in infected B cells. Nevertheless, over the long-term ex vivo cultivation such aberrations appear. In the case of MM, the malignant transformation is inevitably accompanied by chromosomal aberrations, which are always being detected during the karyotyping. Despite these aberrations affect multiple chromosomal loci [17] the most frequent and common for MM pathogenesis ones can be distinguished. First of all, MM cells are aneuploid. The most frequent karyotypic aberrations in the MM cells are disturbances in the integrity of the first chromosome (1p,q), deletion of either the 13th chromosome or its arm (13q) and deletion of 17p. The additional arm of the first chromosome is being detected in 35–40% of total cases and leads to disfunctioning of the *CKS1B*, *ANP32E, BCL9* and *PDZK1* oncogenes. Deletion of the 1p arm occurs in 30% of MM cases, disrupting the functions of *FAM46C*, *FAF1*, and *CDKN2C*. Loss of the 13th chromosome or its arm 13q causes the loss of *RB1* tumor suppressor and occurs in 50% of MM cases. Deletion of the 17p arm leads to the loss of *TP53* tumor suppressor gene and is being characteristic for 10% of MM cases. Other more or less frequent karyotypic aberrations in MM cells include the deletions of 11q (7%, *BIRC2, BIRC3*), 14q (38%, *TRAF*3), and 16q (35%, *CYLD, WWOX*) arms [17–19].

Totally, the findings [7] and the present study indicated that the obtained cell line most likely belongs to EBV-induced lymphoblastoid ones. Thus, the karyotype of these cells was presumed to be either intact or having chromosomal aberrations irrelevant to those most common for MM. To clarify this presumption, a number of FISH assays aimed to detection of MM-specific chromosomal aberrations (TP53, IGH rearrangements, deletions of 13th chromosome or its loci, and aberrations of the first chromosome) has been performed. The obtained results testify the absence of MM-associated chromosomal abnormalities.

Thus, the analysis of the cell line obtained from an aspirate from a patient with MM and capable of forming freely floating spheres indicates that this cell line appears to be a lymphoblastoid cell line. It, in turn, means that this cell line is a progeny of an EBV-infected immortalized B clone that supplanted the primordial clonotypic MM cell during the long-term in vitro cultivation. If the proposed concept is correct, then the vast majority or even all cells of the line should carry the same VDJ locus.

### Identification of the cells from the culture and xenograft as belonging to the dominant clonotypic B-cell clone

In order to clarify the presence of the clonotypic B cell and to prove the relation of the resulting xenograft to the original cell line, the following approach was developed. There is a method for detecting monoclonality (“deep sequencing” variation). A mature B cell has undergone VDJ recombination. It was presumed that if a certain amount of clonotypic B cells would be present in the tumor, then a specific PCR product corresponding to the rearranged VDJ genotype could be obtained using specific primers described in studies on detection of monoclonality [15, 16, 20–23]. Further, B cell validation will include quantification of DNA with this VDJ genotype, which will reflect the number of clonotypic B cells in the resulting xenograft.

**Fig. 2:**
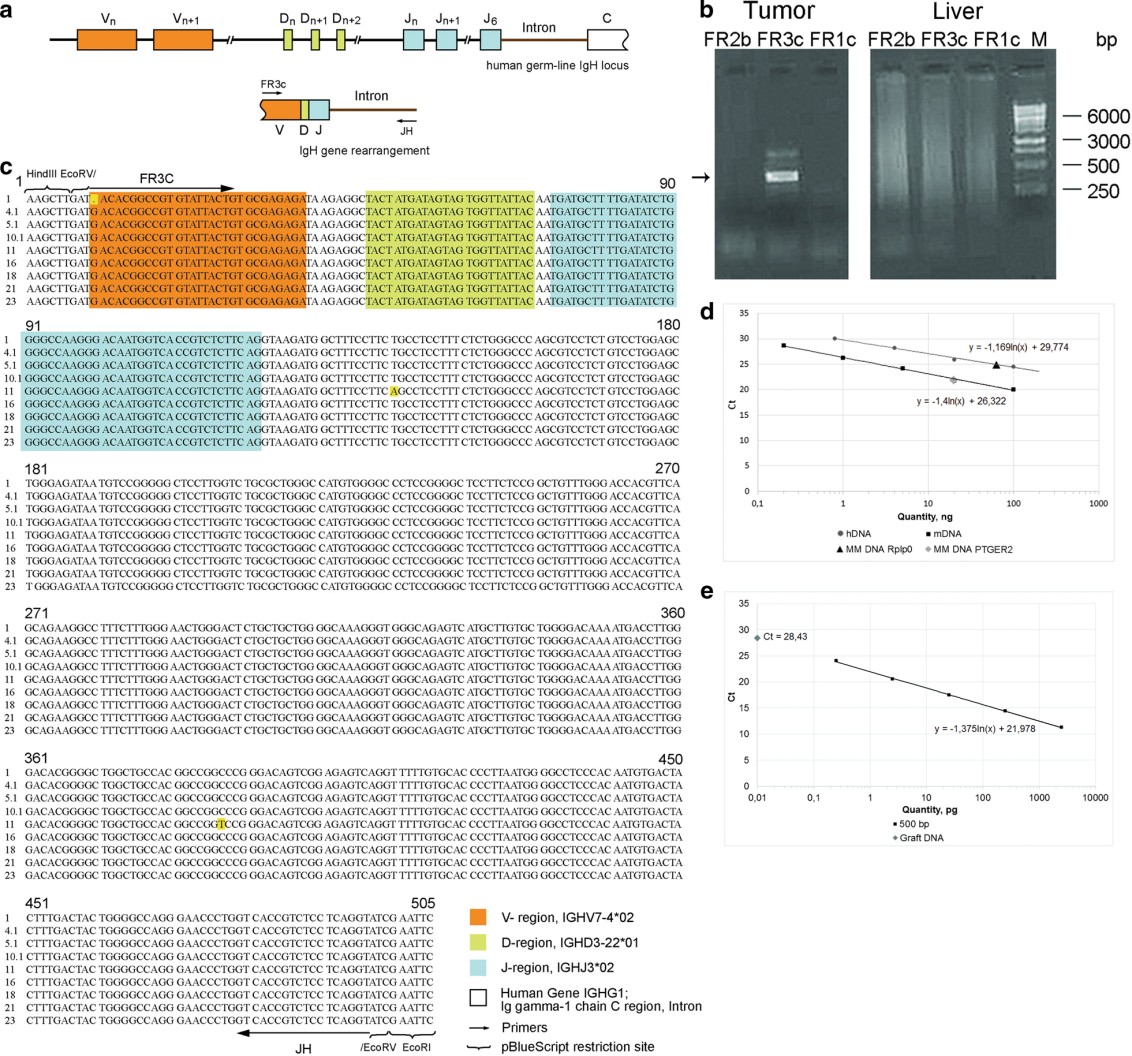
Evaluation of the number of clonotypic B cells with the rearranged VDJ locus in the xenograft. **a** A scheme of VDJ locus differentiation during recombination. **b** PCR. Tumor—PCR with three sets of degenerate primers (JH in each case, and FR2b, FR3c, and FR1c) for conservative regions of the VDJ locus with DNA isolated from the tumor graft. Liver—DNA isolated from the liver of mice; the presence of the fragment indicates the presence of many identical B clones in the DNA sample. **c** Sequencing of the rearranged B clone. Comparison of the sequences of several sequenced clones (figure shows 9 out of 23). The V, D, and J segments are shown in colors corresponding to those in Scheme A. The arrow indicates the primer sequence. The brackets indicate the site for cloning into the plasmid PUC19 (at the *Eco*RV site). The sequences are identical. Individual substitutions are shown in yellow. **d** qPCR with DNA isolated from the tumor graft. The plot shows calibration curves for human DNA (Rplp0 primers, total human DNA as a template) and mouse DNA (PTGER2 primers, DNA from mouse bone marrow cells as a template). Dots on the calibration curves indicate the amount of different DNAs in the tumor graft. Human DNA—67.6 ng, mouse DNA—22.0 ng. **e** qPCR quantification of B clone copies in DNA isolated from the tumor graft. The calibration curve was obtained by titration of B clone DNA (500 bp) and JH/FR3c primers. The dot denotes the number of B clones in studied DNA, which is calculated using the calibration curve slope

We synthesized three pairs of primers for typical regions of the VDJ locus to find the optimal conditions for producing the PCR product (see “Sequencing of VDJ locus from DNA isolated from the xenograft and initial culture” section). The conducted assay resulted in the specific fragment of ~ 500 bp (hereinafter referred to as “500”) (Fig. 2a, b). The fragment was cloned, and several of the resulting clones with the same electrophoretic mobility were sequenced. The sequences of several of these fragments are shown in Fig. 2c. These sequences turned out to be identical, excepting a few single substitutions. The resulting major PCR product and the sequencing results indicated that DNA isolated from xenograft material contained numerous fragments with a similar DNA sequence. However, such experiments always face the issue of selective primer binding aimed to the only one certain DNA molecule, which leads to amplification of only the one PCR product instead of all present. For this reason, we conducted experiments to quantify the content of different DNA types in the xenograft using real-time PCR. The amount of mouse and human DNA was estimated by comparing results of qPCR with specific primers for *PTGER2* gene locus in the case of mouse DNA, and for the *Rplp0* gene locus in the case of human DNA (Fig. 2d). DNA isolated from the tumor graft turned out to contain 67.6 ± 4.1% of human DNA and 22.0 ± 2.2% of mouse DNA. The amount of DNA homologous to the cloned 500 fragment corresponding to the rearranged VDJ locus was evaluated by comparison with the normalization curve obtained for the purified 500 fragment (Fig. 2e). We found that 100 ng of DNA isolated from the xenograft contained 0.009 pg of B clone-specific DNA. The number of cells carrying the rearranged VDJ locus was calculated as follows. The amount of DNA (100 ng) used in the reaction is equivalent to ~ 8.3 × 10^3^ cells (assuming that 1 diploid eukaryotic cell contains 12 pg of nuclear DNA). The amount of B clone-specific DNA (0.009 pg) produced in qPCR corresponds to ~ 11.9 × 10^3^ copies of a 500 bp double-stranded DNA fragment. The diploid cell contains two copies of this DNA fragment. Therefore, the sample of the total xenograft DNA contains the amount of DNA corresponding to 5.9 × 10^3^ clonotypic B cells, which constitutes ~ 71% of the calculated total number of cells. The conducted comprehensive assessment suggests that the xenograft material of the human origin is most likely represented by clonotypic B cells only.

Another issue that had to be answered was identification of the clonotypic B cell in the analyzed spheres. Presence of clonotypic cells with identical VDJ locus in absolute majority in both cellular systems would be the evidence that the developed subcutaneous xenograft resulted from the reported cell line. Thus, finding of the previously identified cells of the B clone in spheres would complete the identification and characterization of the tested biological model.

**Fig. 3:**
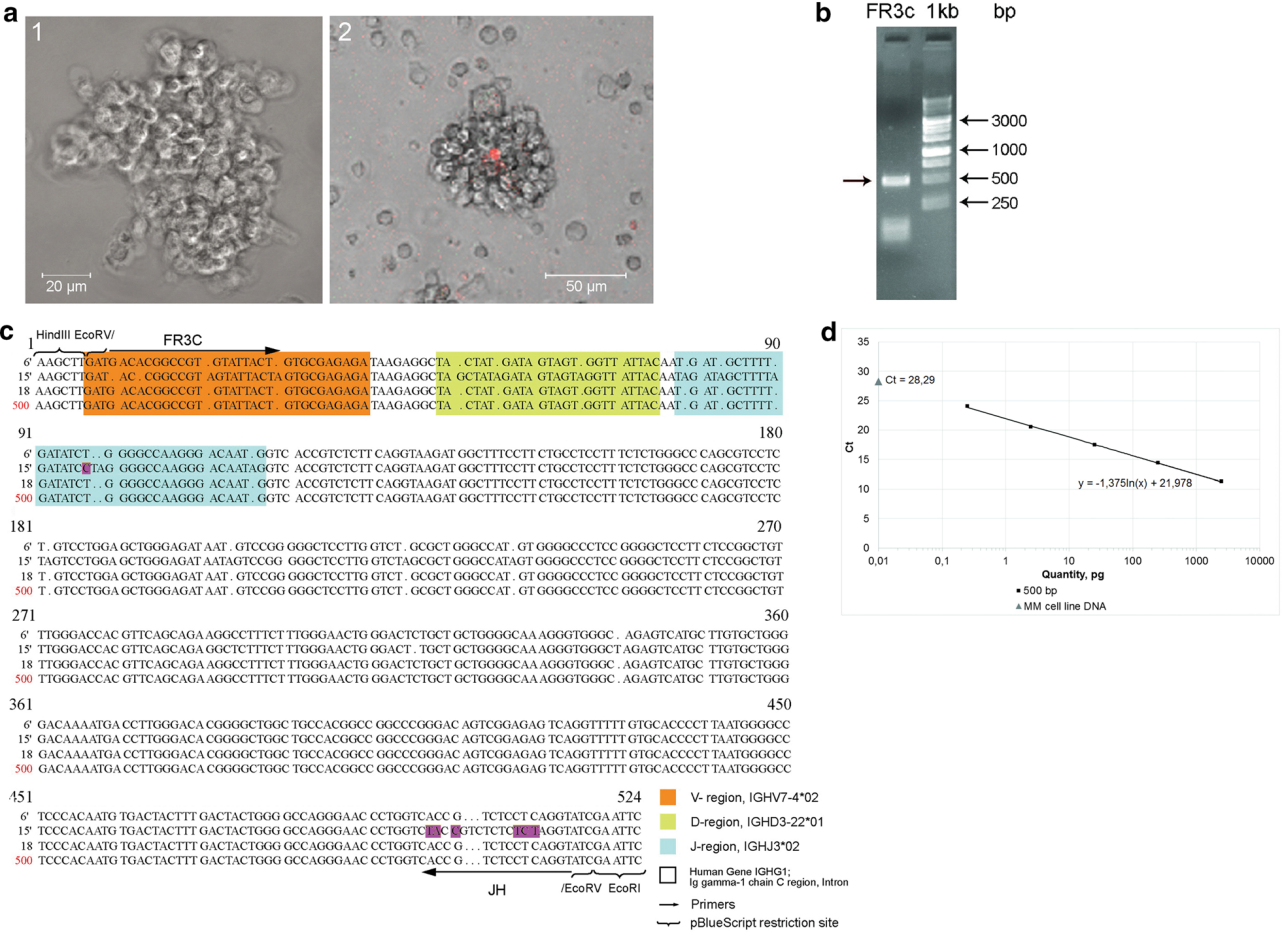
Evaluation of the number of clonotypic B cells with the rearranged VDJ locus in the total number of cells grafted. **a** An image of the sphere in transmitted light (1) and the sphere containing the TAMRA+ cell (2) obtained using a laser scanning microscope. **b** PCR with DNA isolated from the same cells as those used to produce the graft, using JH/FR3c primers. The arrow indicates the 500 bp fragment of the differentiated B clone. **c** Comparison of several clones sequences (3 out of 15) with each other and also with the sequence of the B clone from the graft DNA (500, shown in red). The V, D, and J segments are shown in colors corresponding to those in Fig. 2b. The arrow indicates the primer sequence. The brackets indicate the site for cloning into the plasmid PUC19 (at the EcoRV site). The sequences are identical. Individual substitutions are shown in violet. **d** Quantification of the B clone-specific DNA content in the total DNA isolated from reported cell line. The calibration curve was obtained by titrating purified DNA of the B clone (500 bp) and JH/FR3c primers. The dot denotes the number of VDJ copies in studied DNA, which is calculated using the calibration curve slope

In experiments on identification of clonotypic B cells in floating spheres, an approach similar to that developed for detection of the specific VDJ PCR product in a solid graft was implemented. PCR with DNA isolated from the samples of free-floating spheres, using primers for the target 500 fragment, also yielded the 500 fragment. This fragment was cloned, and several of the resulting clones were sequenced. The results of analysis are shown in Fig. 3a–c. The sequences of the produced clones were identical, excepting a few substitutions, both to each other and to the sequences of clones produced from the PCR fragment synthesized from xenograft DNA. As in the case of DNA from the xenograft, we quantified the content of DNA homologous to the 500 fragment. The amount of this DNA was assessed by comparison with the normalization curve obtained for the purified 500 fragment. Cells sample was shown to contain 0.01 pg of B clone DNA (Fig. 3d). The number of cells carrying the rearranged VDJ locus was calculated using a logical scheme similar to that in experiments with xenograft DNA. The amount of B clone DNA (0.01 pg) obtained in the qPCR experiments corresponded to 13.2 × 10^3^ copies of a 500 bp double-stranded DNA fragment, which are contained in 6.6 × 10^3^ cells, which in turn constitutes ~ 79.4% of the calculated total number of cells. Thus, the clonotypic B cells make up about 80% of the total cellular number in the floating spheres.

The obtained results allow to draw two principal conclusions. First, the xenograft develops from cells of the reported cell line, and second, cells in both biological systems are the progeny of the clonotypic B cell carrying the identically rearranged VDJ locus.

### Clarification of the possible relation of TAMRA-positive cells to three types of stem cells present simultaneously in the bone marrow of the MM patient

Investigating the interplay of eukaryotic cells and extracellular double-stranded DNA, we discovered and characterized two unknown common biological phenomena. We found that: (i) extracellular double-stranded DNA fragments are internalized by undifferentiated cells of different genesis, including tumor-initiating stem cells; (ii) being internalized, double-stranded DNA fragments participate in the reparative process in such a way that interfere its natural implementation. In this case, depending on the type of reparative process or its phase, the involvement of extracellular DNA in repair can either facilitate correct completion of the reparative process (save the stem cell from apoptotic death) or disrupt the correct progression of repair (induce aberrant repair of damaged chromatin), which is accompanied by apoptosis and elimination of the stem cells [7–13, 24, 25].

As shown in the previous study on the topic [7] and in Figs. 3a-2, 5a, and 6a, free-floating spheres derived from the aspirate of a MM patient contain TAMRA+ cells. These cells secrete the specific set of chemokines and, being in aggregation with several unidentified sphere cells, participate in the formation of a sphere-organizing center during the re-aggregation of cells into a spherical 3d structure. These characteristics could mean that TAMRA-positive cells are undifferentiated cells with clonogenic properties.

In the bone marrow, two cell types with similar clonogenic properties are commonly detected: CD34 hematopoietic stem cells and CD90 mesenchymal stromal cells. Cells of both types could be in the reported cell line as a result of contamination. Moreover, this cell line is substantially consisted of clonotypic B cells that have supplanted the primordial cells of MM. We analyzed whether TAMRA+ cells belonged to any of these types of cells.

To clarify the possible relation of TAMRA+ cells to hematopoietic progenitors or to MSCs, the typing of sphere-composing cells with antibodies against CD34 and CD90, respectively, was performed. For CD90-specific antibodies, staining was conducted simultaneously with the detection of TAMRA-DNA probe-positive cells in a single sample.

In addition, a FICTION analysis (combined FISH with a 500 DNA probe and typing with CD90 antibodies in a single sample) was used to clarify the possible relationships between the clonotypic B cells and the MSCs.

Using the real-time PCR with DNA from FACS sorted TAMRA+ cells as well as FISH with the 500 DNA probe combined with TAMRA− (Flu−) DNA internalization in a single slide, we obtained results demonstrating no relation of TAMRA+ cells to the clonotypic B cell.

### Analysis of TAMRA-positive cells with regard to their possible relation to CD34 hematopoietic cells, CD133 cancer cell, CD20 B lymphocytes

In the initial experiments, we paid a significant attention to typing of surface markers of free-floating sphere cells. In the experiments, no CD34-positive cells were detected in free-floating spheres. Below is Table 1 of surface markers, which characterize cells of primary free-floating spheres. The obtained result means that TAMRA+ cells are not CD34 hematopoietic stem cells.

**Table 1:** The phenotype of mesenchymal stromal cells and sphere cells, which is expressed as a percentage of cells carrying an appropriate surface marker

	Mesenchymal stromal cells	Sphere cells
Stromal markers		
CDD73	64.98	≤ 32.21
CD90	98.41	1.13
CD105	87.89	
CD29		10.66
Cancer stem cell markers		
CD44		≤ 89.35
CD133		≤ 3.05
Hematopoietic stem cell marker		
CD34	0.07	0
Macrophage markers		
HLA DR	0.78	93.24
CD14	0.88	5.07
CD16	1.13	
T cell markers		
CD3	0.67	2.03
CD4		0
B cell markers		
CD19		0.27
CD20	0.88	≤ 94.28

Initial cells of the adherent fraction had an almost classical phenotype of mesenchymal stromal cells: CD105+, CD90+, CD73+, CD34−, CD3−, CD20−, HLADR−, CD16−, and CD14−. According to the International Society for Cellular Therapy (ISCT), mesenchymal stromal cells are CD73−, CD105−, and CD90-positive and HLA-DR-, CD45-, CD34-, CD19- or CD79α-, and CD14- or CD11b-negative. Results of the phenotypic analysis of spheres is presented in Table 1. It was established that during the cultivation, the phenotype of spheres has changed as a consequence of primordial MM cell line degeneration associated with EBV infection. As a result, the ratio of surface markers has also changed and currently corresponds to the values indicated below. 1.56% of CD133+/CD20+ cells and 6.03% of CD73+/CD20+ cells were found among sphere cells. In addition, we performed co-typing of sphere cells for their ability to internalize the TAMRA DNA probe as well as for the CD133 cancer cell stemness marker and the CD20 B cell marker [7]. CD20 is specific for B lymphocytes. It is also present on the surface of malignant cells in most B cell lymphoproliferative diseases, except MM. The following ratios were found: TAMRA+ cells, ~ 6%; TAMRA+ CD20+, 4.43%; TAMRA− CD20+, 12.5%; TAMRA+ CD20−, 2.02%; TAMRA− CD20−, 81.05% (Additional file 1: Fig. S1A); TAMRA+ CD133+, 5.1%; TAMRA+ CD133−, 0.60%; TAMRA− CD133+, 0% (Additional file 1: Fig. S1B). Noticeably, almost 90% of TAMRA+ cells carry the CD133 marker, prominin, characteristic of cancer stem cells of several cancer types [26–29]. And finally, since ~ 4.5% of cells are simultaneously positive both for the TAMRA+ marker of undifferentiated cells and for the CD20 marker of B cells, it means that almost all TAMRA+ cells carry the marker of B lymphocytes.

#### Analysis of the relation of TAMRA+ cells to CD90+ (mesenchymal stem) cells

Previously, [12], we demonstrated that the culture of mesenchymal cells contained about 8% of TAMRA+ cells. Also, MM cells are known to have close relationships with MSCs [30–32]. In this regard, it was necessary to assess the possibility for TAMRA+ cells (as possibly related with MM) to be a side population of MSCs. To elucidate if this is actually the case, we conducted experiments on the co-localization of a TAMRA DNA probe and antibodies against the CD90 antigen, a conventional marker for MSCs. Two series of experiments were performed: (i) fluorescence microscopy of cell samples; (ii) dual localization of both markers directly in the ex vivo culture.

**Fig. 4:**
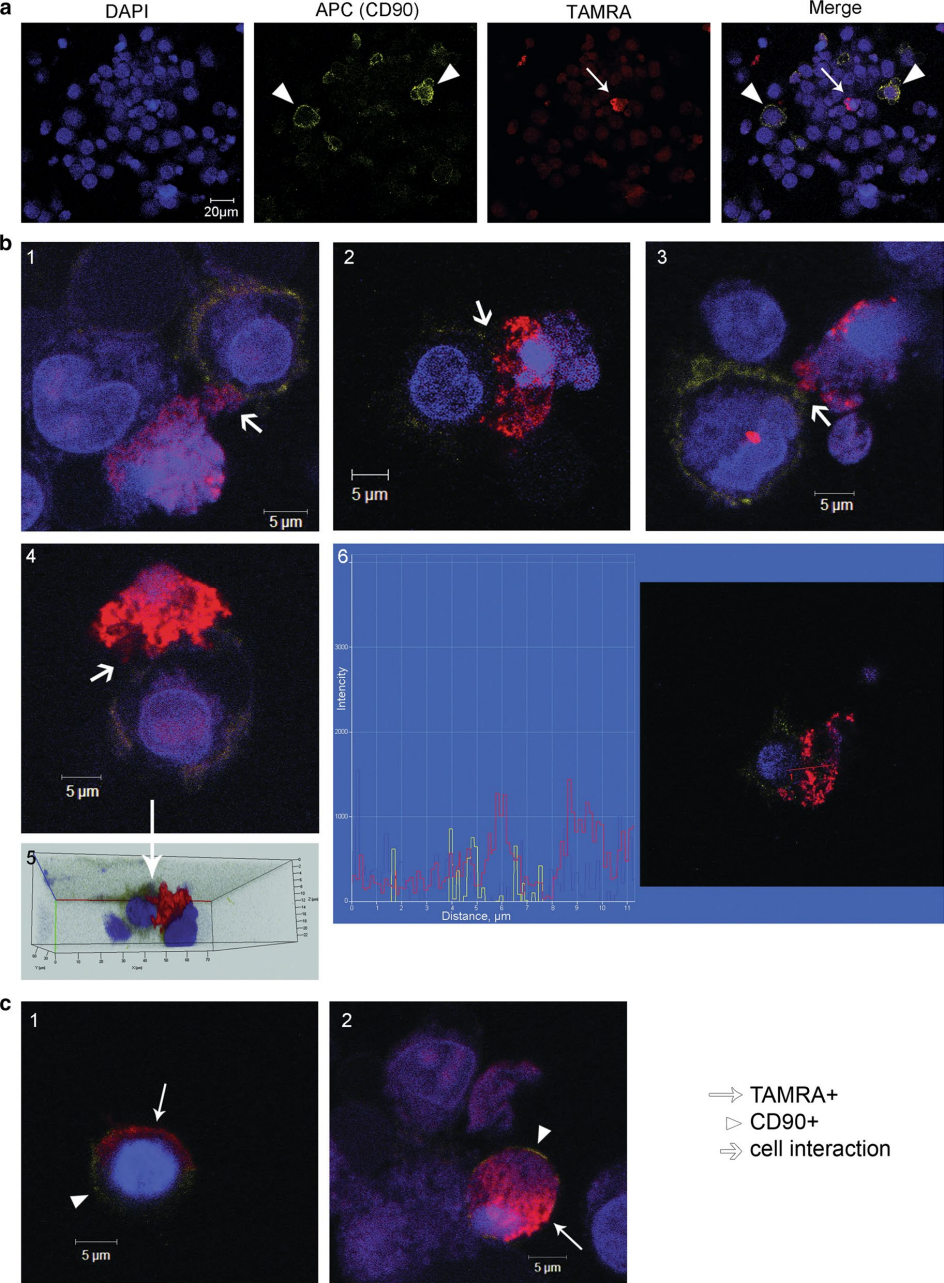
Dual analysis of the cells with regard to their ability both to bind antibodies to the surface marker CD90 and to internalize TAMRA-DNA in cytological preparations. **a** A general view of the dual analysis of CD90+ cells and TAMRA+ cells in a single preparation. **b** Images characterizing the transfer of cellular material from TAMRA+ cells to mesenchymal stem cells (1–4); 5—3d structure of cells interaction presented on 4th panel; 6—fluorescence intensity profile of two interacting cells, red and yellow graphs correspond to the TAMRA and APC (CD90) signals. **c** Cells carrying simultaneously both color markers. DAPI staining of nuclei. Arrows indicate mesenchymal stem cells (CD90 marker), TAMRA-positive cells, and zones of the cellular material transfer

A microscopic analysis of cell samples demonstrated that both cell types were present in formed spherical aggregates (Fig. 4a). Often, these cells formed tight ectopic contacts (Fig. 4b1–3). In many cases of such contacts, TAMRA material was pulled towards CD90+ cells (Fig. 4b4, 5); in some cases, an intermediate phase of TAMRA material transfer from TAMRA+ cells to CD90+ cells was observed (Fig. 4b6). In addition, samples contained: (i) cells simultaneously positive for both CD90 and TAMRA markers (Fig. 4c1); (ii) CD90+ cells with a part of TAMRA+ material localized either at the periphery or in the nuclear space (Fig. 4c2).

**Fig. 5:**
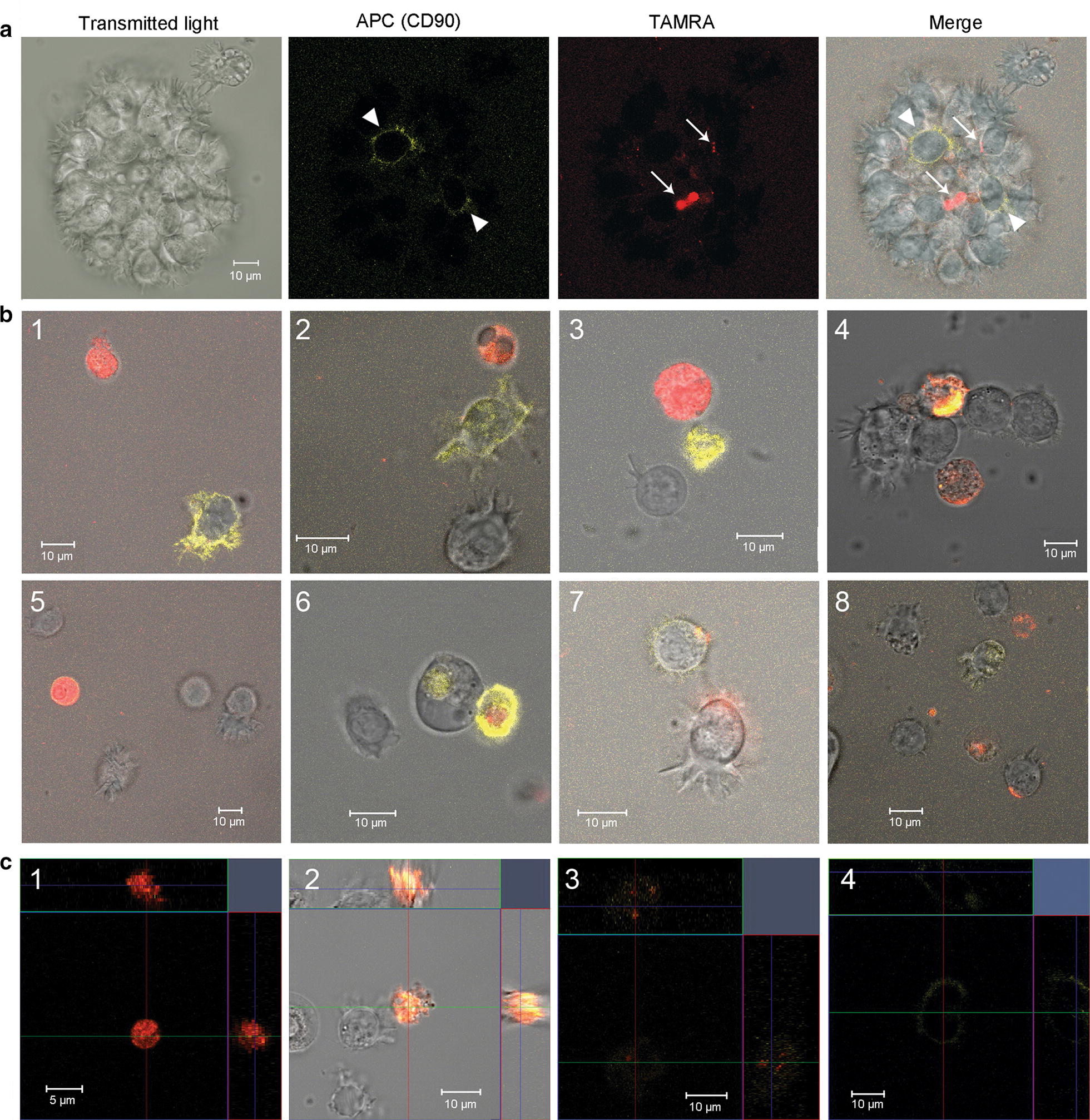
Dual analysis of cells with regard to their ability both to bind antibodies to the surface marker CD90 and to internalize TAMRA-DNA in ex vivo culture. **a** Spheres. Arrowheads indicate CD90+ cells, arrows—TAMRA+ cells. **b** Individual cells. **c** Orthogonal projections of cells showing 3d distribution of the signal in different cells

The results of fluorescence microscopy were further added with the results of co-staining of cells in an ex vivo culture using two markers. The sphere-forming cells were co-treated with anti-CD90 antibodies and TAMRA-labeled DNA for 1 h. These experiments confirmed the results of cytological sample analysis, indicating that cells of both types were present in the formed spherical aggregates (Fig. 5a).

Experiments on the co-localization of a TAMRA DNA probe and CD90 antibodies performed in quintuplicate revealed the localization pattern of the analyzed markers, the respective cell types, as well as their behavioral peculiarities.

The following variants of cells carrying the tested markers were frequently found in analyzed samples. (1) Small cells that captured only the TAMRA DNA probe (intracellular localization) (Fig. 5b1–3, c1). (2) Small cells that captured only anti-CD90 antibodies (intracellular localization) (Fig. 5b3). (3) Small cells that captured both the TAMRA DNA probe and anti-CD90 antibodies (intracellular localization) (Fig. 5b4, c2). (4) Small cells that were labeled with anti-CD90 antibodies at the periphery (cytoplasmic membrane) and captured the TAMRA DNA probe (Fig. 5b5, 6, c3). (5) Large cells with mobile pseudopodia (MSCs), labeled by anti-CD90 antibodies at the periphery (cytoplasmic membrane) (Fig. 5b1, 2, 8, c4). (6) Large cells with mobile pseudopodia (MSCs), labeled by anti-CD90 antibodies at the periphery, having TAMRA material residues either at the periphery or in the inner space of the cell (Fig. 5b7). (7) Large cells with mobile pseudopodia, without the CD90 marker, with TAMRA material residues either at the periphery or in the inner space of the cell (Fig. 4b3, 5b8).

**Fig. 6:**
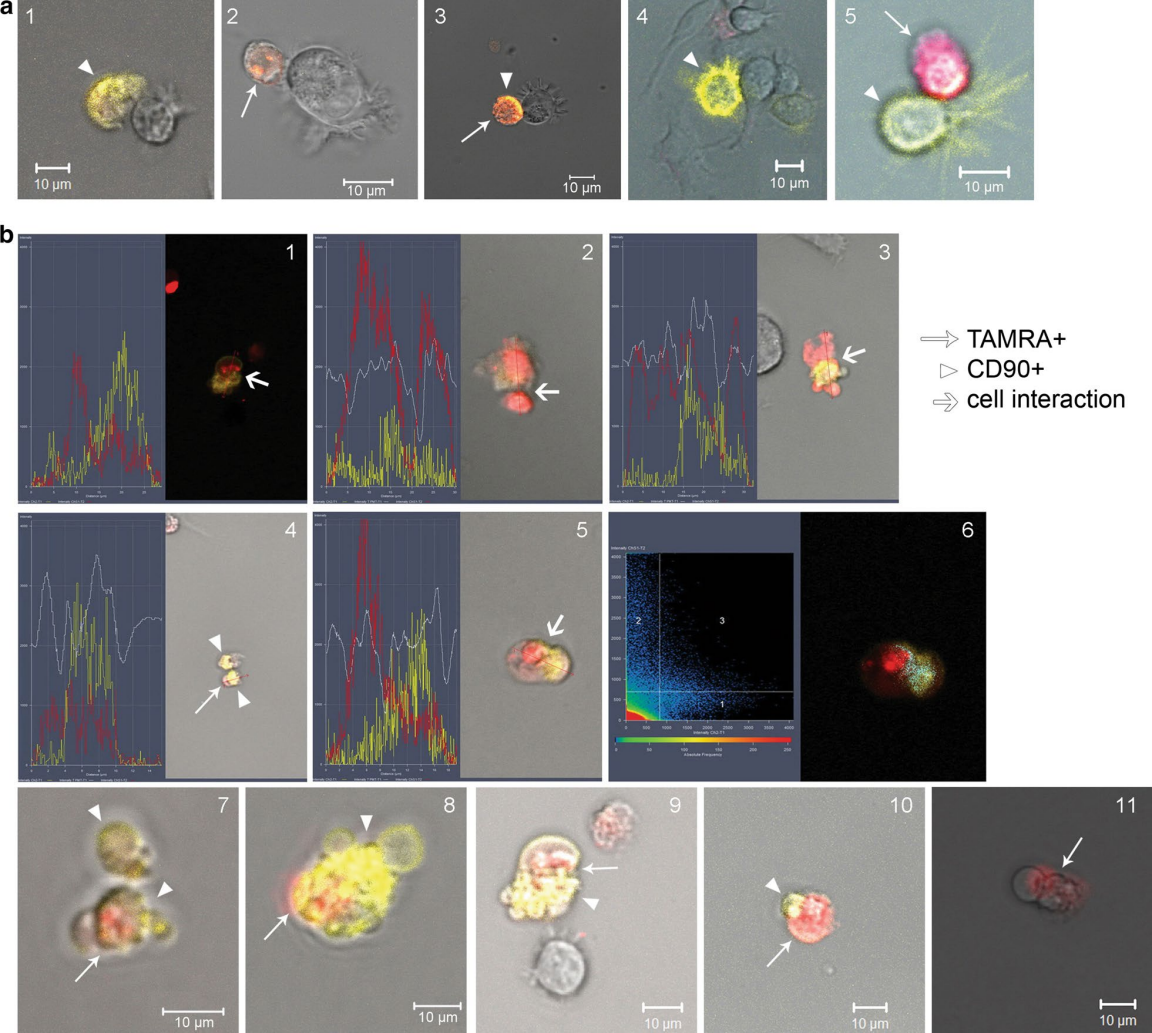
Dual analysis of cells with regard to their ability both to bind antibodies to the surface marker CD90 and to internalize TAMRA-DNA in ex vivo culture. **a** Different cells in direct contact. **b** Confluent cells. 1–5—Fluorescence intensity profiles (CD90 (APC)—yellow line, TAMRA—red line), and a transmitting light profile (white line) built across the interacting cells; 6—APC and TAMRA fluorescence co-localization profile. The site with equal intensity of both signals is denoted in blue; 7–11—images of confluent cells obtained in vitro using the laser confocal microscope

The following cell behavior variants in the analyzed culture were found. (1) Cells contacting each other. All possible combinations of such cells were found. Here they are: CD90+ cells interacting with unidentified cells (Fig. 6a1), TAMRA+ cells in contact with unidentified cells (Fig. 6a2), double-labeled (CD90+/TAMRA+) cells interact with unidentified cells (Fig. 6a3), CD90+ cells with motile pseudopodia interacting with unidentified cells (Fig. 6a4) and CD90+ cells contacting with TAMRA+ cells (Fig. 6a5). Pictures of the general image splitted into separate channels (transmitting light, APC (CD90) and Rho (TAMRA)) are in the supplementary data (Additional file 1: Fig. S2). (2) Fusing cells. Only the cells with intracellular location of markers were found to be fused. In Fig. 6b(1–6) there is a set of images showing the fusion of TAMRA+ cells with cells containing CD90 intracellularly. Noticeably, in some cases this process was accompanied by the formation of numerous vacuole-like structures and appeared as “bubbling”. Figure 6b(7–10) represent four different images of this “bubbling”. The fusion of two TAMRA+ cells were also found (Fig. 6b11).

It should be noted that co-presence of both analyzed markers in one small cell may be associated with two independent processes: internalization of two types of labeled molecules in one cell or fusion of two cells containing different markers. The experimental design presumes the possibility of both variants.

In addition, contacts formation between CD90+ (mesenchymal stem) cells and TAMRA+ cells in real time was monitored (Additional files 2, 3). Two presented videos demonstrate two types of interactions between CD90+ cells (large cells labeled with antibodies at the periphery, with mobile pseudopodia) and TAMRA+ cells. CD90+ cells have actively moving pseudopodia at one end. The opposite end forms ectopic contact with the TAMRA+ cell. In the first case, long-term retention of the TAMRA+ cell by the CD90+ MSC and capture of TAMRA+ material (occurring at the ectopic contact during retention) by the MSC are observed. The moment of dissociation of cells and their re-association is clearly seen. The re-association occurs at the site of previous ectopic contact where residual TAMRA material transferred to CD90+ cell structures is detected. Throughout retention, the CD90+ cell moves by means of mobile pseudopodia that disappear at a certain time, after which the CD90+ cell is rounded and remains in re-association with the TAMRA+ cell (Additional file 2). In the second case, we observe the “search-like” behavior of the CD90+ MSC with active pseudopodial movement and capture of the TAMRA+ cell. There is an attempt to capture the second TAMRA+ cells by pseudopodia. After a certain time of contact, TAMRA material in the form of small spheres is transferred to dendrites formed by the CD90+ (mesenchymal stem) cell (Additional file 3).

The performed analysis suggests the following interpretation of the obtained results. The cell line obtained from a MM patient contains two types of cells positive for these markers. Cells of the first type are small and capable of internalizing a clearly detectable amount of both extracellular DNA (fluorochrome-labeled TAMRA DNA probe) and extracellular proteins (CD90 antibodies). Large cells of the second type form dynamic pseudopodia-like structures and bind anti-CD90 marker antibodies at the periphery, which characterizes them as CD90+ MSCs. Both types of cells positive for the used markers can interact with each other in any combination. Interaction of two or more small TAMRA+ cells may result in a complete fusion, while contacts of TAMRA+ cells with CD90+ MSCs are limited to partial transfer of cytoplasmic content from TAMRA+ cells to CD90+ mesenchymal stem ones.

**Fig. 7:**
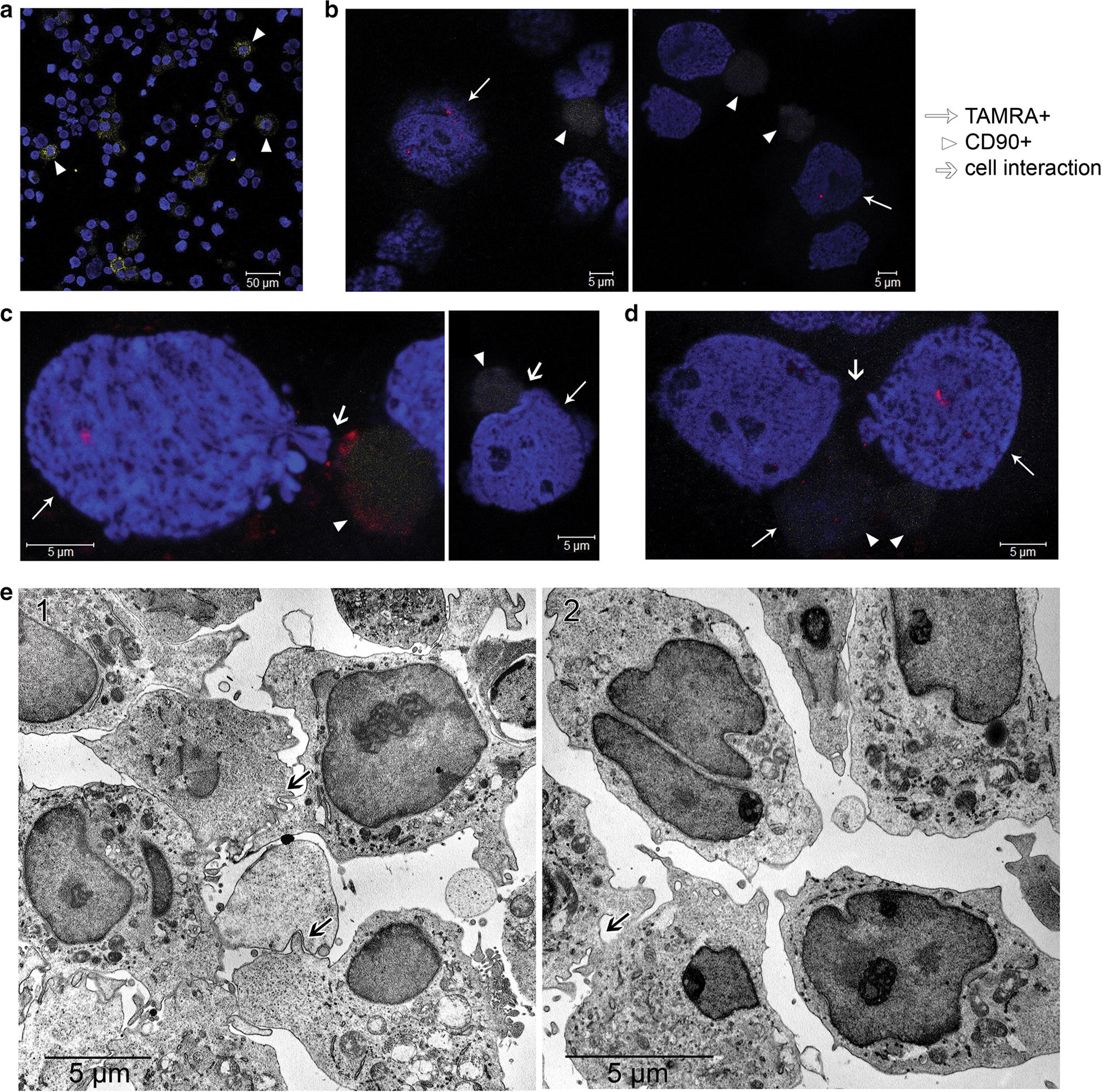
Cells interaction analysis. **a** A cell preparation treated with CD90 antibodies before hybridization. **b**–**d** FICTION analysis. Cell preparations treated with CD90 antibodies and hybridized with the 500 TAMRA-labeled DNA fragment corresponding to the rearranged VDJ locus of the clonotypic B cell. DAPI staining of nuclei. Arrows indicate specific hybridization sites, mesenchymal stem cells (CD90 marker), and areas of ectopic contacts of clonotypic B cells and mesenchymal stem cells. **e** Transmission electron microscopy analysis. 1—Typical contact interactions between cells in sphere structure are shown by arrows. In these cases, one of the cells contacts the neighboring cells through cytoplasmic processes. 2—Group of cells with a typical morphology: increased nuclear-cytoplasmic ratio, presence of two nuclei in individual cells, high euchromatin content in nuclei, polar non-uniform distribution of organelles in the cytoplasm. The site of cell membranes fusion in the form of cytoplasmic bridges is shown by arrow

In addition, the interplay between the clonotypic B cell and the CD90+ cell were characterized by fluorescence microscopy (Fig. 7a–d). Among others, two rarely observable, and thus being of particular interest, patterns of interactions between these cells were discovered. First, most of the cells carrying any of these marker fluorochromes are located independently of each other. However, we detected cells carrying both the CD90 antigen and characteristic hybridization signals (Fig. 7d). In addition, we found some peculiar ectopic contacts between the cell with classical hybridization signal and the cell labeled with anti-CD90 antibodies (Fig. 7c, d). Finally, ectopic contacts between cells, both in spherical structures and in a single state, were confirmed by electron microscopy (Fig. 7e). The obtained data are in agreement with the described phenomenon of active ectopic interaction accompanied by a potential exchange of cellular information between CD90+ MSCs and other cells in vitro.

#### Clarification of the possible relation of TAMRA-positive cells to clonotypic B cell

**Fig. 8:**
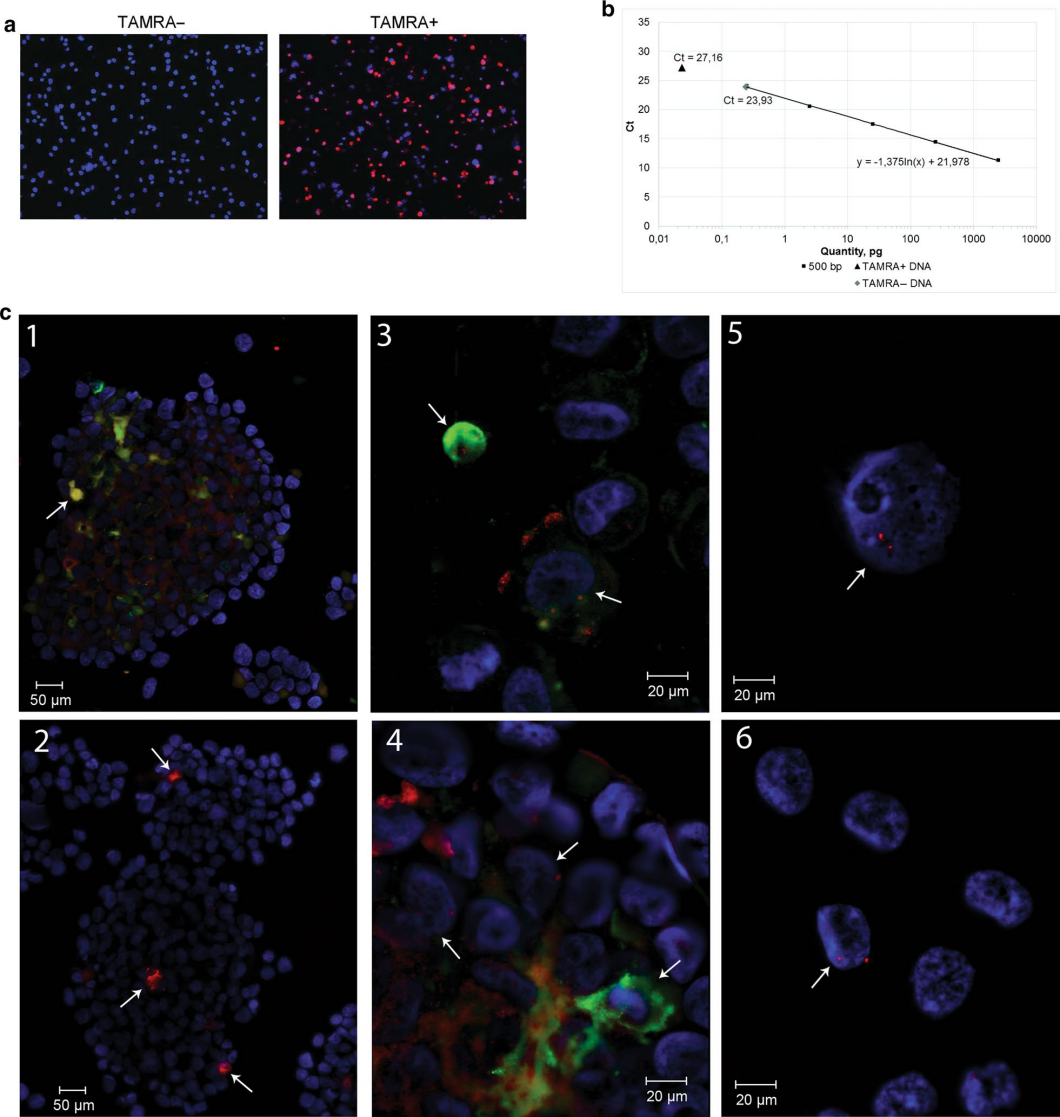
Analysis of the relation of TAMRA-positive cells to the clonotypic B cell. **a** Evaluation of the efficiency of cell sorting based on DNA internalization (*Alu*-TAMRA). **b** qPCR quantification of the B clone DNA (500 bp) content in sorted TAMRA+ and TAMRA− cells. The calibration curve was plotted using titration of purified B clone DNA (500 bp) and JH/FR3c primers. Dots denote amounts of the fragment in TAMRA+ and TAMRA– DNA. **c** Preparations of cells after internalization of the Flu or TAMRA *Alu*-DNA probe followed by FISH in a single preparation with the TAMRA-labeled 500 DNA fragment corresponding to the rearranged VDJ locus of the clonotypic B cell. 1, 2—Intact spheres with the Flu (1) and TAMRA (2) *Alu*-DNA probe. 3, 4—Sphere fragments. 5, 6—Cells of the fraction of free cells and destroyed sphere fragments. Arrows indicate the sites of specific hybridization of the probe’s DNA and cells captured the fluorochrome-labeled DNA probe. DAPI staining of nuclei

In the final part of our study, we conducted experiments clarifying the TAMRA+ cells relation to clonotypic B cells of EBV+ LCL (Fig. 8).

Free-floating sphere cells were disaggregated and treated with TAMRA-labeled *Alu* DNA. After treatment, the cells were separated into two TAMRA+ and TAMRA− populations (Fig. 8a). After this, DNA was isolated from both samples, and quantitative real-time PCR was performed. The analysis results are shown in Fig. 8b. The idea of the experiment was as follows. If TAMRA+ cells were of clonotypic B clone, then the same or similar (depending on the number of these cells) amount of clonotypic VDJ DNA would be detected upon a quantitative assay of the respective sample. Conversely, the PCR product should not be detected in the TAMRA− sample. In the conducted experiments, the following numerical values were obtained. In TAMRA+ and TAMRA− samples, the content of B clone-specific DNA was 0.023 pg and 0.24 pg, respectively, out of total 100 ng in the reaction. Based on 4 independent qPCRs, the content of B cells-specific DNA in TAMRA− sample was ~ 10.5 ± 4.1-fold higher than that in TAMRA+ sample. In additional experiments, cells of both types were tested using combined FISH with the 500 DNA probe and TAMRA (or Flu) internalization in a single preparation (Fig. 8c). No overlapping fluorescence was found. Taken together, these findings indicate that clonotypic B cells and TAMRA+ cells exist as two different and independent populations.

## Discussion

The thorough scrutiny of the reported cell line with regard to its origin and congruence with the MM was conducted.

- The line was found to be contaminated with EBV. It is known that, being contaminated with EBV, MM cell lines are not capable of the long-term existence [6].
- The significant majority of cells in the line (more than 93%) are CD20-positive. MM cells are known to be CD20-negative [6].
- Cells of the reported line carry no chromosomal aberrations similar to those distinguishing of MM.
- The doubling rate of the reported cell line is approximately 48 h. In the case of MM cells, this parameter is about 108–144 h [4].
- The reported cell line in its majority is consisted of clonotypic B cells with identically rearranged VDJ locus. Cells of this line do not secrete any class-specific immunoglobulins. Similarly, MM cell lines are also consisted of the dominant clonotypic cells, but these cells, in contrast to cells above, obligatory secrete the class-specific immunoglobulin (IgEL).
- The reported cell line was shown to form the large and tight spheres and their aggregates. MM cell lines occasionally form tightly aggregated small conglomerates of cells [4].
- The reported cell line is capable of xenograft formation free of allogeneic stromal matrix, but only upon the whole spheres transplantation. Spheres, being dispersed to single cells, failed to produce a xenograft in all three transplantation attempts [7]. MM cell lines are capable of xenograft development only upon the allogeneic stromal matrix formation [33, 34].

Taking all the data obtained, it is clear that the reported cell line is the EBV+ LCL, resulting from the expansion of the dominant EBV-infected B-cell clone, which completely replaced the primordial MM cells during the long-term (over 3 years) cultivation. Since this cell line has acquired the status of immortalized one, and is being routinely used in our investigations, it was given the HH47 name. Currently, the HH47 cell line is under registration in the official list of the cell lines repository of the Institute of Cytology and Genetics SB RAS. The conducted experiments indicate that the clonotypic B cells with the identical VDJ locus constitute the absolute majority both in the produced xenograft and in the xenograft-producing cell line. This fact testifies the xenograft origin from the reported HH47 cell line.

One of the characteristics of the reported cell line is its ability to form free-floating spheres. Spheres are cell conglomerates formed due to the adhesive properties CD90+ MSCs as well as to the chemoattractive effect of chemokines secreted by TAMRA+ and TAMRA− cells [7]. The majority of sphere is constituted of monoclonal B cells. The evaluated number of clonotypic LCL B cells in the sphere sample is 79.4%. Thus, the main participants in the sphere formation process are the cells of three types: TAMRA+ cells, CD90+ MSCs and clonotypic B cell. It is this set of cell types that allows the HH47 cells to continuously form 3d spherical structures in vitro.

The necessity of TAMRA+ cells in sphere aggregation was demonstrated in additional experiments with sorted TAMRA+ and TAMRA− cells. In TAMRA− population, aggregation is started 6 h later than in control sample or upon adding the TAMRA+ cells to TAMRA− population (Additional file 1: Fig. S3). Moreover, the formed cells associations are significantly smaller and appear to be a stochastically aggregated cells instead of separately floating large spheres of classic spherical shape (Additional file 1: Fig. S4).

The question is how the presence of three types of clonogenic cells in the investigated line could be explained? Presence of MSCs in the primary culture could be due to adhesion of MM cancer cells to bone marrow stromal cells mediated by the MM-specific protein, CS-1 [30]. During the establishment of this cell line, MM cells and mesenchymal stromal cells attached to the plastic surface either independently of each other or being adhered to each other via CS-1. As a consequence, the reported cell line had been contaminated with these MSCs.

Expansion of the dominant EBV-positive B-cell clone has resulted in the elimination of MM cells, while MSCs remained as a functional subpopulation in the culture. The ability of the cell line to form spheres emerged coincidingly with its metamorphosis due to domination of the EBV-infected B-cell clone. It is the lymphoblastoid B cells that are capable of forming spheres, while the monoclonal MM cells are always not [4].

Not being clonotypic B cells, TAMRA+ cells of the reported cell line carry CD133 marker, produce specific chemokines and, as mentioned, are involved in the formation of a sphere-organizing center [7] and seem to be essential participants of the bone marrow niches.

In the bone marrow, there are few small populations of differentiated cells, partially sharing specific markers, which form bone marrow niches (PDGFRα+ CD51+ Nestin+ cells, PDGFRα+ Sca-1+ PαS cells, PDGFRα+ CD51+ Scf+, LepR+ cells) [35]. These cells could be related to the subpopulation of TAMRA+ cells present in a small proportions (less than 1%) in the mouse and human bone marrow [11]. However, as follows from our previous reports, the ability to capture extracellular double-stranded DNA fragments is the hallmark of only undifferentiated cells, both normal and cancerous [7–14]. All the mentioned indicates that TAMRA+ cells have no relation to these low-numbered subpopulations of the bone marrow cells.

Cancer stem-like cells (CSCs) were discovered in the late 90s [36, 37]. Similarly to normal stem cells, CSCs are featured with their capability of dividing asymmetrically and maintaining a pluripotent status indefinitely [38–42].

Nevertheless, the most important feature of CSCs, which distinguishes them from normal stem cells fundamentally, is their metastasizing capability that allows these cells to form the stromal microenvironment suitable for further development of tumor regardless of the initial cellular environment. This attribute is primarily associated with an ability of CSCs to retain a pluripotency outside the specific niches while being resistant to stemness-suppressing influence of normal tissue stroma [43], and determined by changes in expression of genes responsible for epithelial-to-mesenchymal transition [44–46].

The clonotypic B cell belongs to a particular type of cancer cells and has some features of CSCs: unrestricted proliferative potential, multiple drug resistance, and the ability to efflux a lipophilic dye and express a variety of ALDHs. However, the clonotypic B cell is supposed to originate from a differentiated B cell [33, 47–50]. Therefore, the existence of CSCs for B-cellular lymphomas in terms of a concept describing CSCs as a small population of undifferentiated cells at the top of a hierarchical pyramid of malignant neoplasms remains controversial. In this context, the presence of TAMRA+ cells in a total cellular mass of the reported cell line could be explained as follows. These cells were initially obtained with MM aspirate and survived the cultivation process as being mandatory for the formation of tumorigenic associations of EBV-immortalized terminally differentiated lymphocytes, originally lacking tumorigenic potential. Displaying the stem-like properties, TAMRA+ cells are proposed to be of bone marrow stromal origin and determine the tumorigenicity of spheres upon transplantation. The indicated peculiarities imply these cells as a kind of “niche-forming” clonogenic MM helper cells participating in aggregation of lymphoblastoid spheres, which are functionally similar to bone marrow stromal MM niches and are necessary for the existing of EBV+ lymphoblastoid cells in vitro.

Two findings in the current study were surprising: direct transfer of cellular material from TAMRA+ cells to CD90+ MSCs and fusion of two cells of the same type that captured either TAMRA DNA or CD90 antibodies. There are studies demonstrating the direct contacts between MSCs and other cell types, including cancer cells [51–56], and providing evidence of direct physical transfer of cellular material from one cell to another [53, 57–61], up to complete fusion (cellular cannibalism or entosis) [62, 63]. The mechanisms of these interactions are almost unexplored. It is known that cellular material can be transferred through formation of microtubular tunnels and occurs in both directions [59, 61, 64]. In the reported cell line, the MSC is an acceptor of TAMRA+ cell material. According to the obtained results, TAMRA+ cells can completely fuse with each other. In this case, a typical “bubbling” of the cell contents occurs. This fusion is typical of embryonic stem cells, embryonic teratocarcinoma cells, embryonic germ layer cells, and somatic cells in vivo and ex vivo [65–70]. Cell fusion can also provide the progression of cancer cells to more malignant and more resistant to therapeutic agents [71, 72]. The previously obtained data and present findings suggest that TAMRA+ cells belong to poorly differentiated cells that for MM aspirate have not been characterized yet.

The discovered phenomena require further research. Also, further research is required to identify the ontological relation of TAMRA+ cells with other bone marrow cells in Multiple Myeloma.

## Conclusions

Initially obtained primary Multiple Myeloma cell culture was experimentally proved to be supplanted after 3-year cultivation by the clonotypic B-cells due to EBV infection. The resulting cell line, being subcutaneously engrafted into NOD/SCID mice, provides the graft development. To produce the graft, the engrafted cells must be aggregated into free-floating spheres, which is the principal hallmark of the reported cell line. The cell line was molecularly, genetically and phenotypically characterized.

The long-term existence of this cell line was established to be due to the presence of three different types of cells with clonogenic properties, namely TAMRA+, CD90+ mesenchymal stem and clonotypic B cells. Sphere-forming capability was shown to be due to direct interaction between TAMRA+ and mesenchymal stem cells. Cells of these two types mandatory present in free-floating spheres as well as form the sphere-organizing center.

## Additional files

**Additional file 1.** EBV+ lymphoblastoid cell line spheres characteristic.

**Additional file 2.** Video demonstrating interactions between CD90+ MSC and TAMRA+ cells.

**Additional file 3.** Video demonstrating “search-like” behavior of the CD90+ MSC with active pseudopodial movement and capture of the TAMRA+ cell.

## Abbreviations

CSCs: cancer stem-like cells
EBV: Epstein–Barr virus
LCL: lymphoblastoid cell line
MM: multiple myeloma
MSCs: mesenchymal stem cells
TAMRA: tetramethylrhodamine fluorochrome
