# Supplementary material for: Identification of the xenograft and its ascendant sphere-forming cell line as belonging to EBV-induced lymphoma, and characterization of the status of sphere-forming cells
Source: Cancer Cell Int. 2019 May 6;19:120. doi: 10.1186/s12935-019-0842-x (PMC6503443; doi:10.1186/s12935-019-0842-x)
Supplement: Supplementary file 1 — Additional file 1. EBV+ lymphoblastoid cell line spheres characteristic. [file 12935_2019_842_MOESM1_ESM.pdf]

Additional file 1

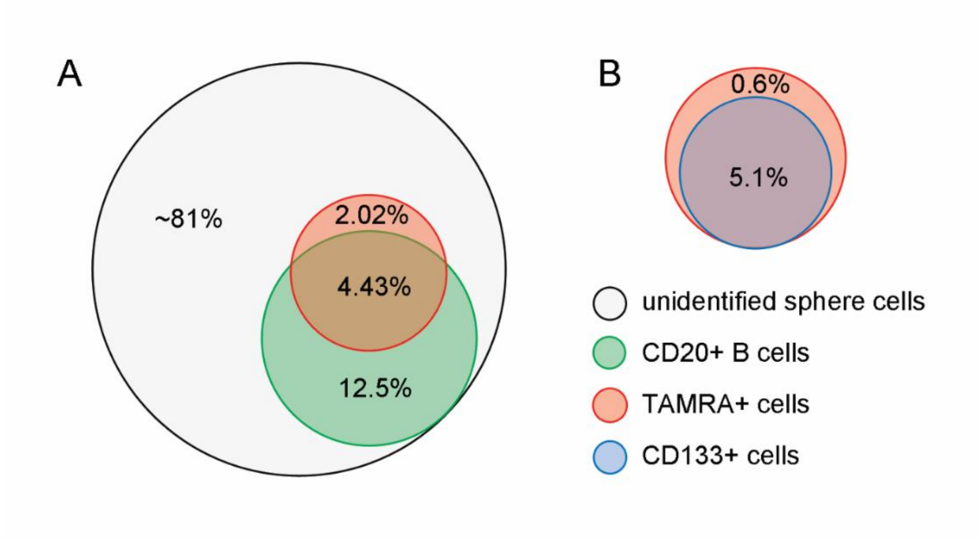

Fig. S1. Schematic representation of the relationship of CD20+, TAMRA+ and CD133+ cells located in the EBV+ LCL spheres.

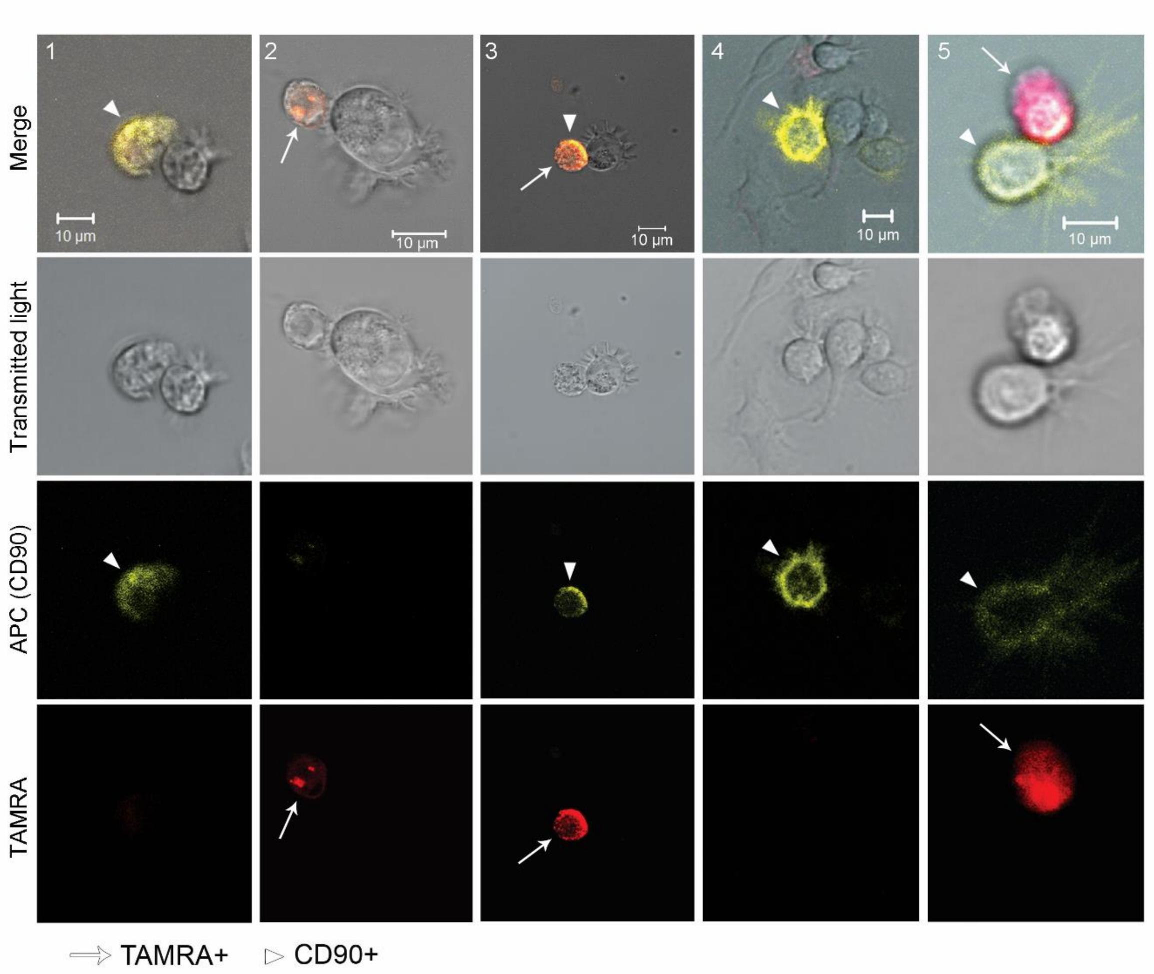

Fig. S2. Dual analysis of cells with regard to their ability both to bind antibodies to the surface marker CD90 and to internalize TAMRA-DNA in *ex vivo* culture. **1-5** – Different cells in direct contact. TAMRA – internalized exogenous DNA signals; APC – marker of CD90+ (mesenchymal stem cells); Merge – combined image of all channels.

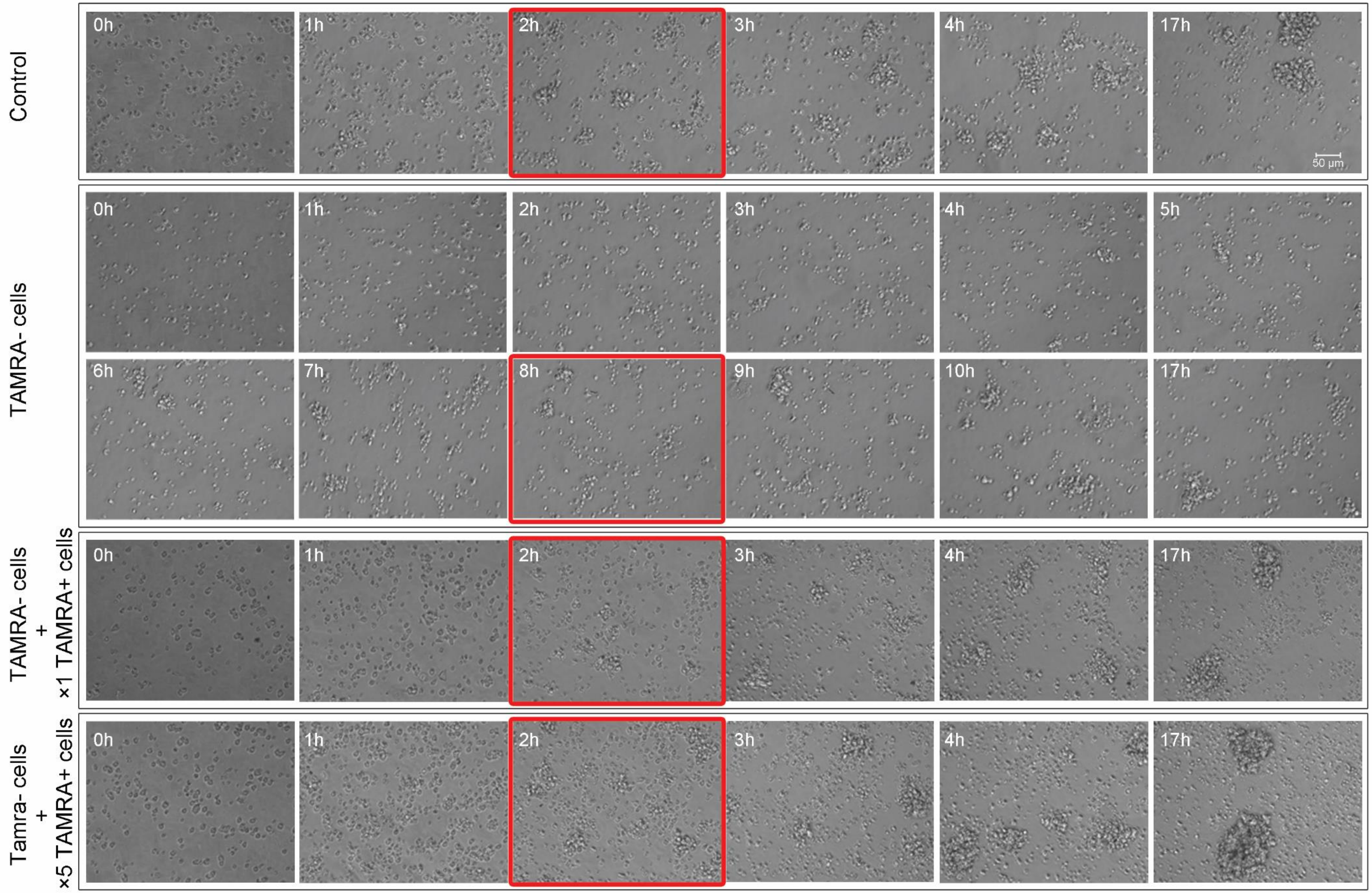

Fig S3 . The rate of sphere formation in EBV+ lymphoblastoid cell line after sorting. The initial stage of cell aggregation, after which spherical 3D structures begin to appear, indicated by a red frame.

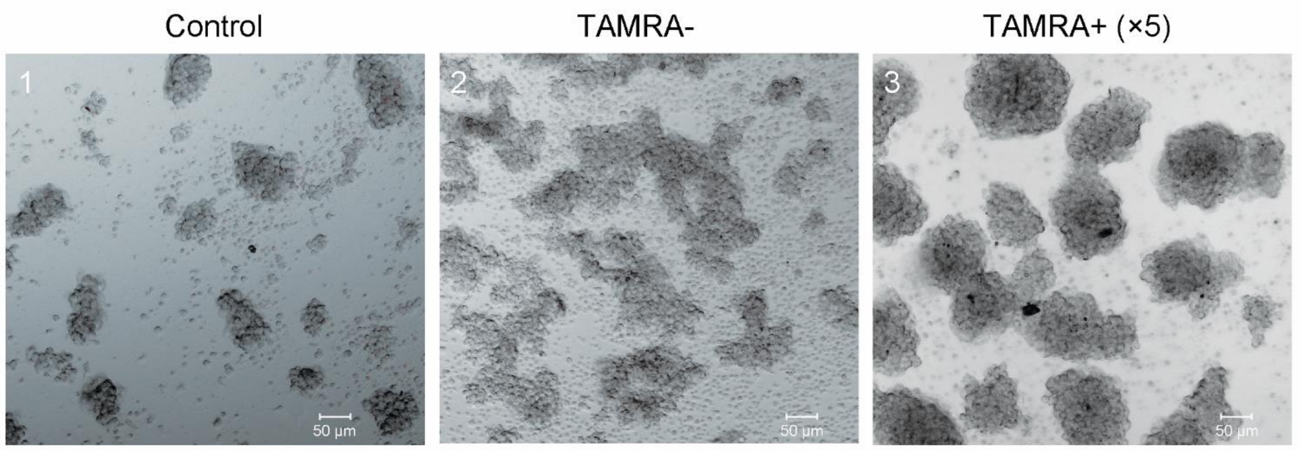

Fig. S4. Variants of sorted EBV+ LCL cells aggregation after 17 hrs cultivation.
